# Protocatechuic acid inhibits aflatoxin production and modulates gene expression in *Aspergillus flavus*

**DOI:** 10.1038/s41598-025-09695-4

**Published:** 2025-07-10

**Authors:** Tarek A. El-Desouky

**Affiliations:** Department of Food Toxicology and Contaminant, National Research Centre, Dokki, Giza, Egypt

**Keywords:** Aflatoxin B_1_ (AFB_1_), *Aspergillus flavus*, Anti-aflatoxins, Inhibition, Protocatechuic acid (PCA), Chemical biology, Microbiology

## Abstract

Aflatoxins, particularly Aflatoxin B_1_ (AFB_1_), produced by *Aspergillus flavus* and other species, pose significant health risks due to their carcinogenic properties. This study investigates the inhibitory effects of Protocatechuic Acid (PCA) on mycotoxigenic fungi and AFB_1_ production. PCA demonstrated significant dose-dependent antifungal activity against various *Aspergillus* species, with *A. flavus* showing inhibition zones ranging from 5.3 mm to 16.7 mm at concentrations of 50 µg/ml to 250 µg/ml, while *A. ochraceus* exhibited the highest sensitivity, with zones up to 23.6 mm. Additionally, PCA effectively reduced AFB_1_ production in liquid media, achieving up to 80.21% inhibition at 250 µg/ml, and decreased the mycelial weight of *A. flavus* by 60.8%. Gene expression analysis revealed that PCA significantly downregulated the expression of the AFB_1_ biosynthetic genes *nor*-1 (95% reduction) and *omt-A* (74% reduction), suggesting that PCA disrupts multiple stages of aflatoxin synthesis. Furthermore, PCA demonstrated efficacy in controlling AFB_1_ contamination in postharvest corn grains, with inhibition percentages of 44.8%, 55.7%, and 64.6% at 150, 200, and 250 µg/ml, respectively. These findings indicate PCA’s potential as a natural antifungal agent, offering promising applications in food safety and postharvest management.

## Introduction

**Fig. 1:**
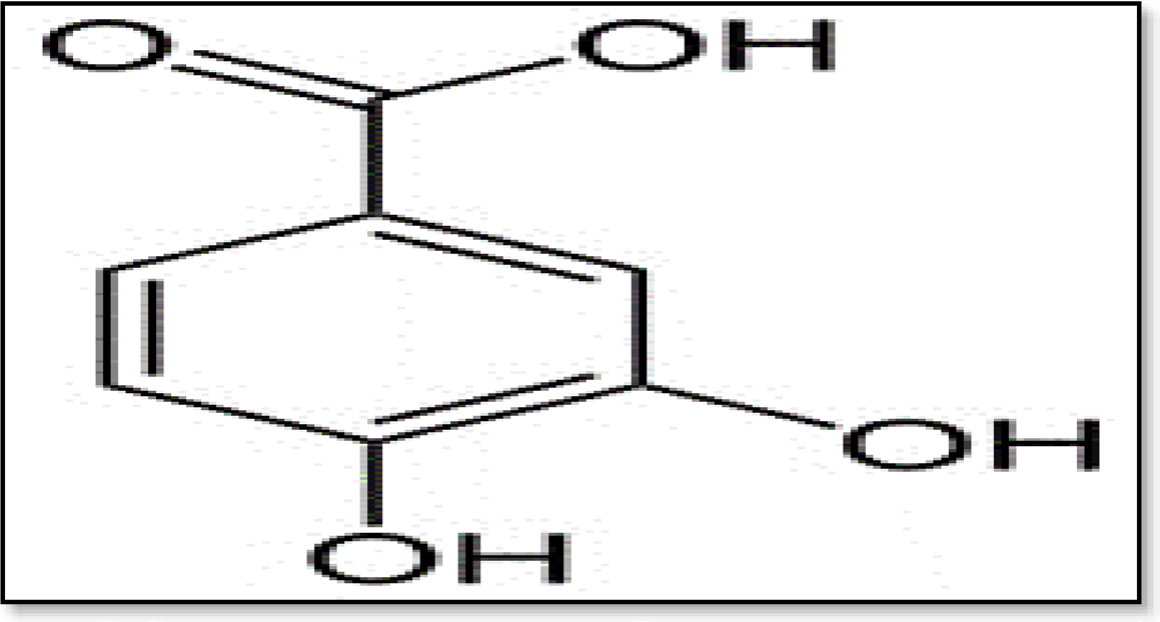
Chemical Structure of protocatechuic acid (C7H6O4).

Aflatoxins (AFs) are a group of secondary metabolites produced by various *Aspergillus* species, including *Aspergillus flavus*, *A. parasiticus*, *A. nomius*,* A. bombycis*, *A. pseudotamarii*, and *A. aflatoxiformans*^1,2^, which are formed under specific conditions such as temperature and relative humidity during pre-harvest, postharvest, transportation, and storage^3^. There are four primary types of AFs: B_1_, B2, G_1_, and G_2_, with AFB_1_ being particularly dangerous. AFB_1_ is known for its carcinogenic, teratogenic, and mutagenic effects on a wide range of organisms and it is linked to liver cancer in humans^4,5^. The International Agency for Research on Cancer (IARC) has classified AFB_1_ as one of the most potent human carcinogens, placing it in Group 1^6^. In response to the health risks associated with aflatoxins, regulatory bodies have set permissible levels for AFB_1_ in food. The European Commission has established a limit of 5 µg/kg for corn, while the FDA allows a higher limit of 20 µg/kg. To manage AFB_1_ contamination in food, both chemical and physical methods are commonly employed; however, these approaches can result in harmful chemical residues and reduced nutritional value. This has led to increased interest in safer alternative methods, including the use of natural and commercial compounds. While synthetic chemicals remain the most widely used solutions, strict regulations and political pressure are reducing their application. Consequently, various naturally derived compounds have been studied for their antifungal and anti-aflatoxigenic activities, many of which have shown great potential in controlling AFB_1_ contamination^7–9^. One such compound, protocatechuic acid (PCA), has the molecular formula C₇H₆O₄ (Fig. 1). It is a naturally occurring phenolic compound found in various fruits, vegetables, and medicinal plants. PCA has gained attention for its antimicrobial, antioxidant, and anti-inflammatory properties^10^. Recent studies suggest that PCA could play a significant role in inhibiting the growth of fungi^11^. To the best of our knowledge, no research has been conducted on the specific mechanisms by which PCA suppresses aflatoxin biosynthesis, which remain unclear. This warrants further investigation to elucidate its potential applications in food safety and AFB_1_ management. This study aims to assess the inhibitory effects of PCA on the production of AFB_1_ by *A. flavus* in liquid media and in white corn infected with this fungus. Additionally, it explores the impact of PCA on the expression of key genes involved in the AFB_1_ biosynthetic pathway, specifically *nor-*1 and *omt-A*, to gain a deeper understanding of the molecular mechanisms underlying its inhibitory action.

## Materials and methods

### Fungal strain

Four toxigenic fungi strains from *Aspergillus* spp were used in this study *A*. *flavus* (ATCC 28542), *A*. *parasiticus* (ATCC 26692), *A. ochraceus* (ATCC 22947) and *A*. *aflatoxiformans*, that deposited to NCBI GenBank with accession # MN093924.

### Chemicals and solvents

Protocatechuic acid (PCA), standard of AFB_1_, potato dextrose agar (PDA) and yeast extract agar were obtained from Sigma-Aldrich, Lyon, France. All solvents were of HPLC grade. The water was double distilled with a Millipore water purification system (Bedford, MA, USA).

### Methods

#### Antifungal activity assays

The fungal suspension was streaked onto PDA, and 5 mm wells were filled with different concentrations of PCA (50–250 µg/mL). Wells with 10% DMSO served as negative controls, while Nystatin (1000 units/mL) was the positive control. The fungal strains were incubated at 28 °C for 3 days, with treatments replicated three times and averaged for results^12^.

#### Assay of anti-aflatoxins activity in liquid media

In this experiment, yeast extract sucrose (YES) culture medium was prepared and autoclaved before inoculating it with a spore suspension of *A. flavus*. Various concentrations of PCA (50, 100, 150, 200, and 250 µg/mL) were added to the medium, which was then incubated at 28 °C for 14 days. After incubation, AFB_1_ was extracted using chloroform, filtered, evaporated to dryness, and analyzed via HPLC, following **El-Desouky**,** 2022** method^13^. The percentage inhibition of AFB_1_ was calculated using a specified equation.

$$\text{The percentage of inhibition AF}\text{B}{_1}=\frac{\mathbf{C}-\mathbf{T}}{\mathbf{C}}{\mathbf{\times100}}$$

where: C is mean concentrations of AFB_1_ in the positive samples that inoculated by spores of *A*. *flavus*. T is concentrations of the AFB_1_ in the sample containing spores of fungus and PCA.

#### RNA extraction and reverse transcription

Total RNA was extracted from the *A. flavus* strain treated with different concentrations of PCA using TRIzol Reagent following the manufacturer’s protocol. RNA quantity and quality were determined using a Spectrostar NanoDrop (BMG Labtec GmbH, Germany) at 260/280 nm absorbance. Approximately 2 µg of total RNA was reverse transcribed into cDNA using Applied Biosystems’ 2X Reverse Transcriptase Master Mix according to the manufacturer’s instructions. All primers were synthesized, and their sequences are listed in Table 1, selected based on the gene sequences available on the GenBank database (http://www.ncbi.nlm.nih.gov/) for quantitative real-time PCR (qRT-PCR).

#### Real-time PCR analysis of aflatoxin biosynthetic genes

The Applied Biosystems 7500 Fast Real-Time PCR system (USA) was used to quantify the mRNA. The quantitative PCR was performed using SYBR Green Master Mix in a 20 µL reaction mixture (TOPreal™ qPCR 2X PreMIX). According to Liang et al. (2015)^14^, the PCR program included an initial denaturation step at 95 °C for 5 min, followed by 40 cycles of denaturation at 95 °C for 25 s, and a final annealing step at 58 °C for 25 s. PCR products were analyzed using the Sequence Detection System Software 1.9.1 (Applied Biosystems). The fluorescence signals were continuously measured once per cycle after the annealing and extension step. Significant differences in gene expression were calculated using the ΔΔCt method (Rao et al., 2013)^15^. Data were standardized with 18 S rRNA, and gene expression between the treatment and control groups was compared.

**Table 1:** Primer used in this study for amplification of AFB_1_ gene.

Target gene	Primer name	5’−3’ nucleotide sequence
*omt-A*	*aflP*- F	GACCAATACGCCCACACAG
	*aflP*-R	CTTTGGTAGCTGTTTCTCGC
*nor-1*	*aflD*- F	GTCCAAGCAACAGGCCAAGT
	*aflD*- R	TCGTGCATGTTGGTGATGGT

### Impact of PCA on the production of AFB_1_ in corn grains

Autoclaved white corn grains were used in five experimental groups, each with 100 g of grains: (1) positive **c**ontrol: Corn grains inoculated with a spore suspension of *A. flavus*; (2) negative control: grains sprayed with PCA only at 250 µg/ml; and (3, 4, and 5) corn grains sprayed with *A. flavus* spore suspension plus 150, 200, and 250 µg/ml PCA, respectively. All groups were incubated for seven days at 28ºC with 85% humidity, with three replicates set for each group. AFB_1_ was extracted using an immune affinity column (Aflatest^®^-P affinity column) and analyzed for concentration using HPLC^16^.

### Statistical analysis

All data were analyzed using the General Linear Model procedure in SPSS version 18. Two-way ANOVA was performed to evaluate the effects of PCA concentration and fungal species on inhibition zones. Assumptions of normality and homogeneity of variances were checked prior to analysis. Statistical significance was considered at *P* ≤ 0.05. Where significant differences were detected, Tukey’s Honestly Significant Difference (HSD) post-hoc test was applied to determine pairwise differences between treatment means. Values are presented as means ± standard deviations from triplicate experiments.

## Results and discussion

### Effect of PCA on growth of mycotoxigenic fungi

**Fig. 2:**
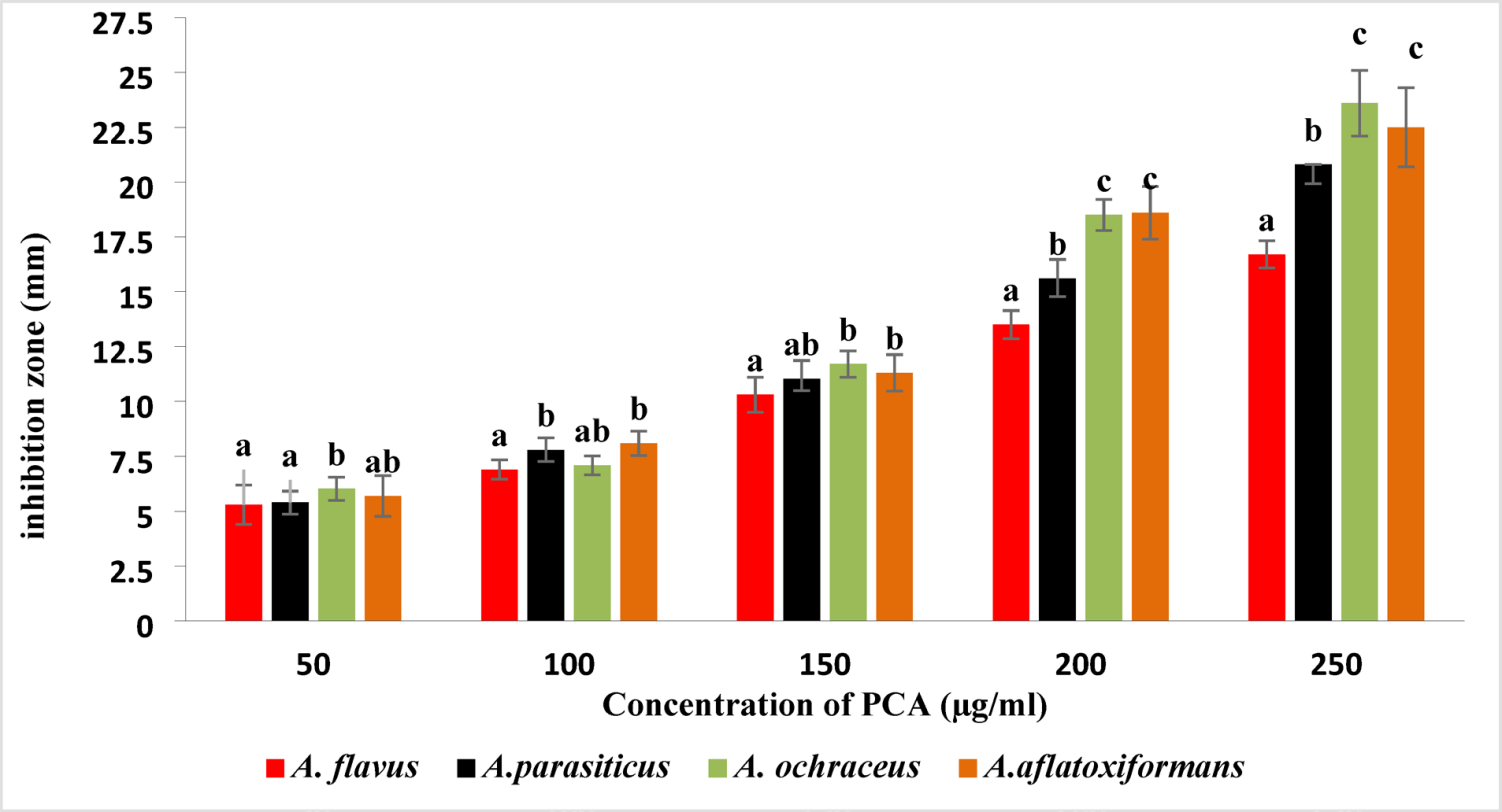
Inhibition zones of different Aspergillus species treated with PCA. Bars marked with different superscript letters (a, b, c) at the same concentration represent statistically significant differences (*P* ≤ 0.05).

Data in Fig. 2 shows the inhibition zones (mm) of different *Aspergillus* species (*A. flavus*,* A. parasiticus*,* A. ochraceus*, and *A. aflatoxiformans*) at various concentrations of PCA ranging from 50 to 250 µg/ml. The data indicate that the highest level of inhibition is detected as the concentration of PCA increases; there is a significant increase in the inhibition zones for all four fungal species. This suggests a dose-dependent antimicrobial activity, meaning that the higher the concentration of PCA, the greater the inhibitory effect on fungal growth. On the other hand, the different fungal species show varying levels of sensitivity to PCA. *A. flavus* exhibits inhibition zones ranging from 5.3 mm at 50 µg/ml to 16.7 mm at 250 µg/ml, indicating moderate sensitivity. *A. parasiticus* shows a more pronounced response compared to *A. flavus*, with inhibition zones increasing to 20.8 mm at 250 µg/ml. *A. ochraceus* shows significant inhibition, with the largest zones observed at higher concentrations (23.6 mm at 250 µg/ml), indicating high sensitivity. Similarly, *A. aflatoxiformans* displays substantial inhibition, with a maximum inhibition zone of 22.5 mm at 250 µg/ml, suggesting a strong response to PCA.

The variation in PCA sensitivity among these *Aspergillus* species can be attributed to several underlying biological and molecular mechanisms. One well-documented antifungal action of PCA involves disruption of the fungal cell membrane through interaction with ergosterol, a critical sterol responsible for maintaining membrane structure, fluidity, and function. Species-specific differences in ergosterol content, biosynthesis efficiency, or membrane composition can influence the extent to which PCA binds to or disrupts the membrane, thereby altering sensitivity^17–19^. Fungal strains with less robust membrane architecture may be more susceptible to PCA-mediated destabilization, leading to leakage of intracellular contents, inhibition of nutrient transport, and ultimately cell death.

In addition to membrane targeting, PCA—although generally recognized as an antioxidant—can also exhibit pro-oxidant activity under certain biological conditions. This dual role is concentration- and context-dependent and is often triggered in the presence of transition metals such as Fe²⁺ or Cu²⁺, which catalyze redox cycling reactions. These reactions can lead to the excessive accumulation of reactive oxygen species (ROS), thereby inducing oxidative stress in the cell. Elevated ROS levels cause oxidative damage to key cellular components, including lipids, proteins, and nucleic acids. The ability of each *Aspergillus* species to tolerate oxidative stress depends largely on the strength of its antioxidant defense system, particularly the activity of enzymes like catalase, glutathione peroxidase, and superoxide dismutase. For instance, *A. flavus* may possess more robust antioxidant mechanisms that can mitigate PCA-induced oxidative stress, resulting in lower growth inhibition. In contrast, more sensitive species such as *A. ochraceus* may lack efficient detoxification systems, making them more vulnerable to ROS accumulation and oxidative damage. This paradoxical pro-oxidant effect of PCA has been previously reported in mammalian cells and supports its context-dependent mode of action^20–23^.

Moreover, the dual nature of PCA—acting as both an antioxidant and a pro-oxidant depending on context—is a key factor in its selective efficacy. In certain systems, PCA scavenges ROS, while in others it enhances ROS production, especially when antioxidant defenses are weak or overwhelmed^24^. This context-dependent behavior likely contributes to the variation in inhibition zones across species. In more vulnerable fungi, PCA can disrupt redox homeostasis, resulting in oxidative damage that leads to mitochondrial dysfunction, loss of ATP synthesis, and impaired spore viability.

Beyond oxidative and membrane-related effects, PCA may inhibit fungal growth by targeting other essential cellular processes. These include suppression of cell wall biosynthesis enzymes, which compromises structural integrity^25^; inhibition of mitochondrial respiration, which reduces ATP production necessary for growth and metabolism^19^; and interference with spore germination, limiting the organism’s reproductive potential^11^. The overall antifungal effect of PCA is therefore multifactorial, involving synergistic disruption of structural, energetic, and reproductive pathways. Importantly, the relative contribution of each mechanism may differ among species. Some fungi may compensate for one mode of action but remain vulnerable to others. For example, *A. flavus* may partially resist oxidative stress but remain susceptible to membrane disruption or mitochondrial inhibition, while *A. ochraceus* may lack resistance across multiple pathways, explaining its higher sensitivity. This broader mechanistic view is consistent with findings from other natural antifungal agents such as cinnamaldehyde, gallic acid, and eugenol, which also exhibit species-specific antifungal profiles. These differences are typically linked to the target organism’s intrinsic oxidative stress response, membrane composition, and gene expression regulation^14,26^. Collectively, these findings highlight the importance of conducting species-specific assessments when evaluating antifungal agents like PCA. They also emphasize that understanding interspecies variation can guide the targeted application of such compounds in food safety and postharvest protection strategies.

Supporting these mechanistic insights, a two-way ANOVA (Table 2) was performed to assess the effects of PCA concentration and fungal species on growth inhibition zones. The analysis revealed highly significant main effects of PCA concentration (F₄,₄₀ = 550.59, *P* < 0.0001) and fungal species (F₃,₄₀ = 30.32, *P* < 0.0001). Additionally, the interaction between PCA concentration and fungal species was significant (F₁₂,₄₀ = 6.02, *P* < 0.0001), indicating that the inhibitory effect of PCA differs among the fungal species tested. Post-hoc comparisons, as shown by different superscript letters in Fig. 2, confirm significant differences among species within each PCA concentration. These results quantitatively support the mechanistic explanations above by demonstrating that PCA’s antifungal activity varies significantly depending on both dose and species.

Finally, it should be noted that the present study provides indirect evidence suggesting that oxidative stress contributes to PCA-induced fungal inhibition. However, direct quantification of intracellular reactive oxygen species (ROS) levels would offer stronger mechanistic validation. In future work, we plan to employ fluorescence-based assays, such as DCFH-DA staining, to measure ROS accumulation in *Aspergillus* species treated with PCA. This approach will enable quantification of oxidative stress responses and correlation of ROS levels with inhibition zones and sensitivity profiles. Such comparative analysis will clarify whether higher ROS accumulation underlies the greater susceptibility observed in species like *A. ochraceus* and *A. aflatoxiformans*, and whether *A. flavus* maintains more effective redox homeostasis under PCA stress. Additionally, we intend to evaluate antioxidant enzyme activities (e.g., catalase, glutathione reductase) to further characterize species-specific detoxification mechanisms.

**Table 2:** ANOVA analysis of variance for effect of PCA on growth of ***Aspergillus spp.***

Source	SS	df	MS	F	*P*
Intercept	9081.090	1	9081.090	10231.849	0.000000
Concentrations of PCA	1954.657	4	488.664	550.588	0.000000
Types of Fungi	80.739	3	26.913	30.324	0.000000
Con. of PCA*Types Fungi	64.110	12	5.342	6.019	0.00000
Error	35.501	40	0.888		
Total	11216.098	60			

SS, sum of squares; df, degree of freedom; MS, mean square; P, probability at confidence 0.95.

### Efficacy of PCA in reducing AFB_1_ production in liquid media

**Fig. 3:**
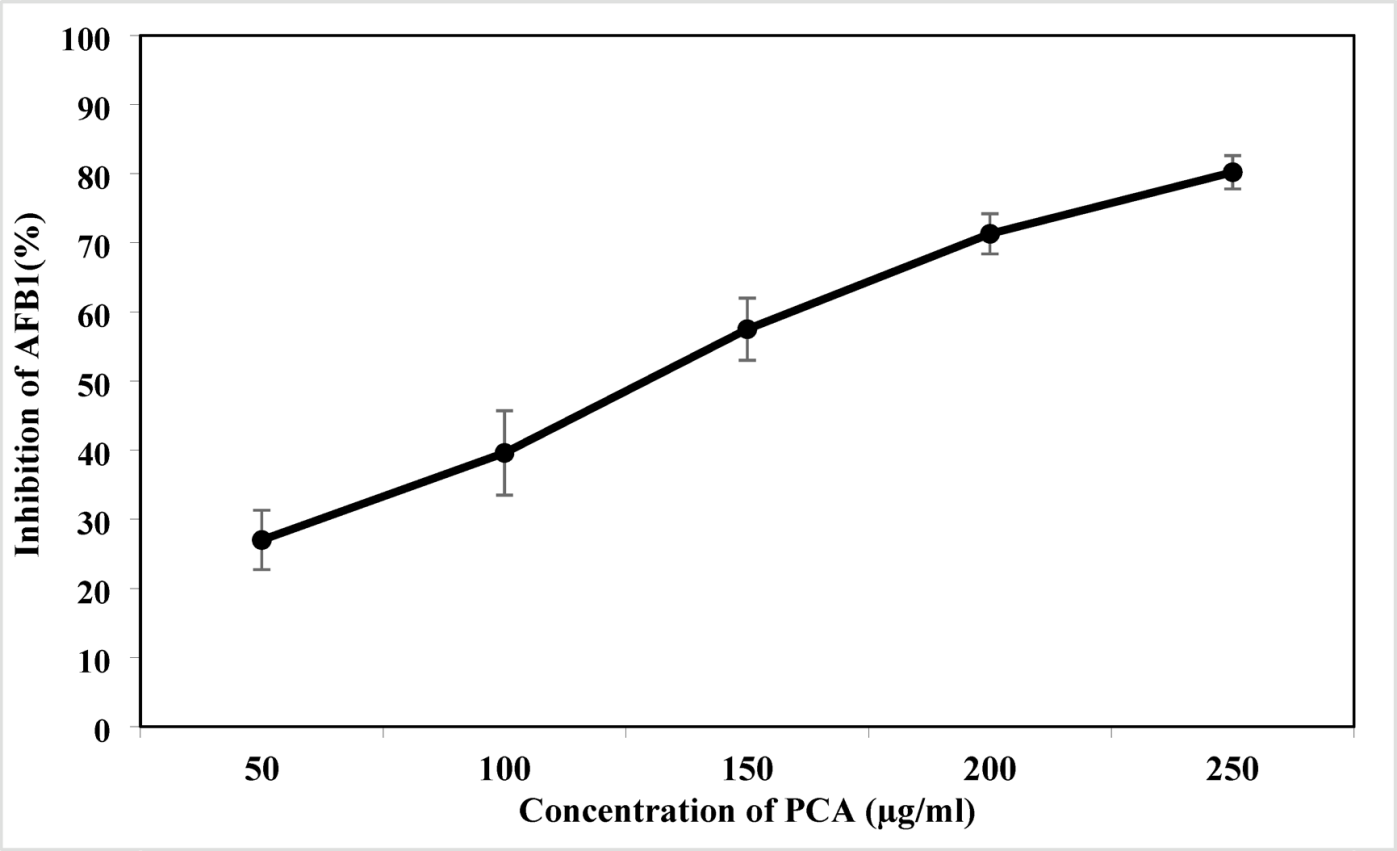
Reduction of AFB1 levels in YES medium following PCA treatment.

**Fig. 4:**
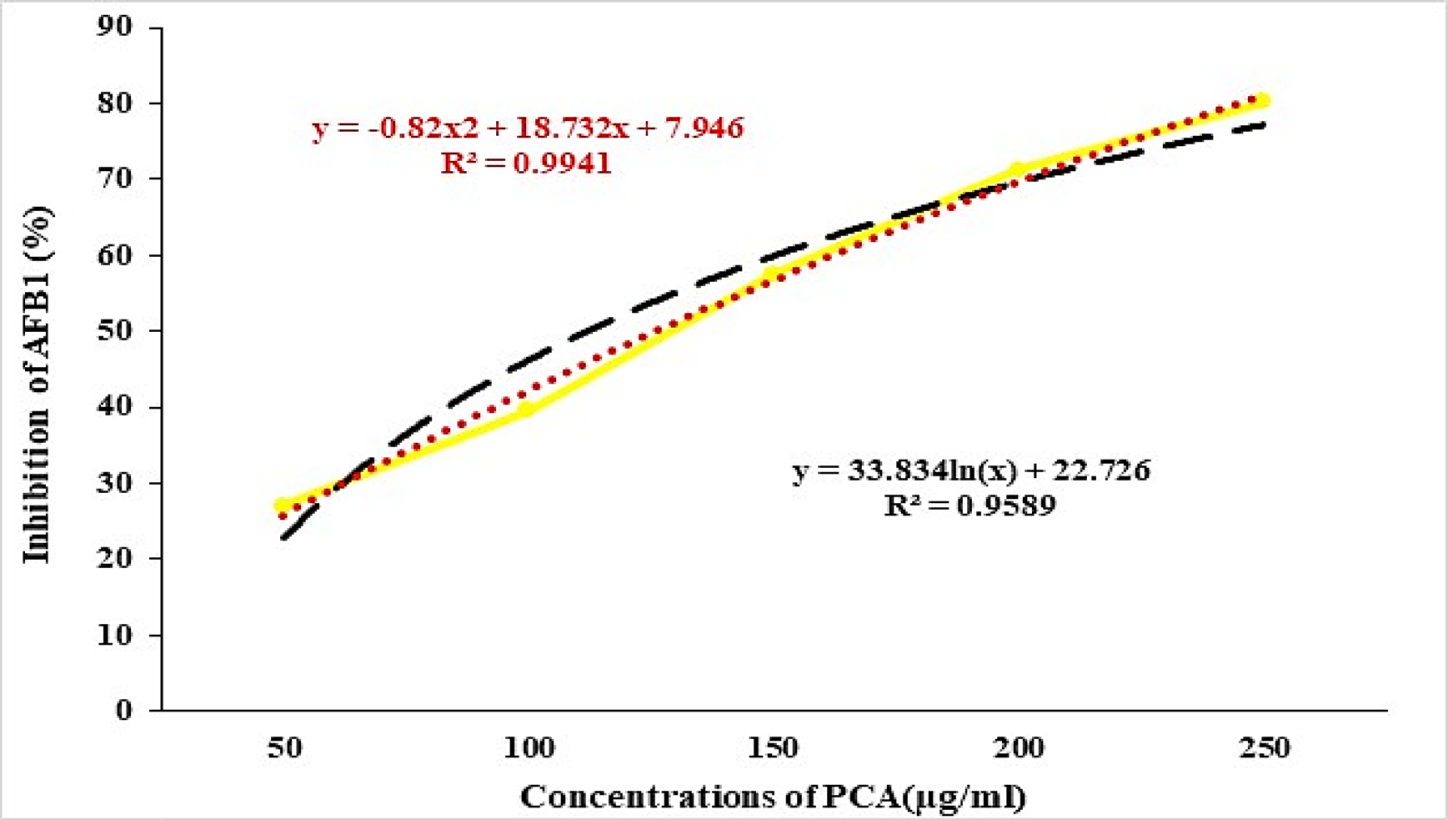
Polynomial and logarithmic regression models for the reduction of AFB_1_ by PCA.

**Fig. 5:**
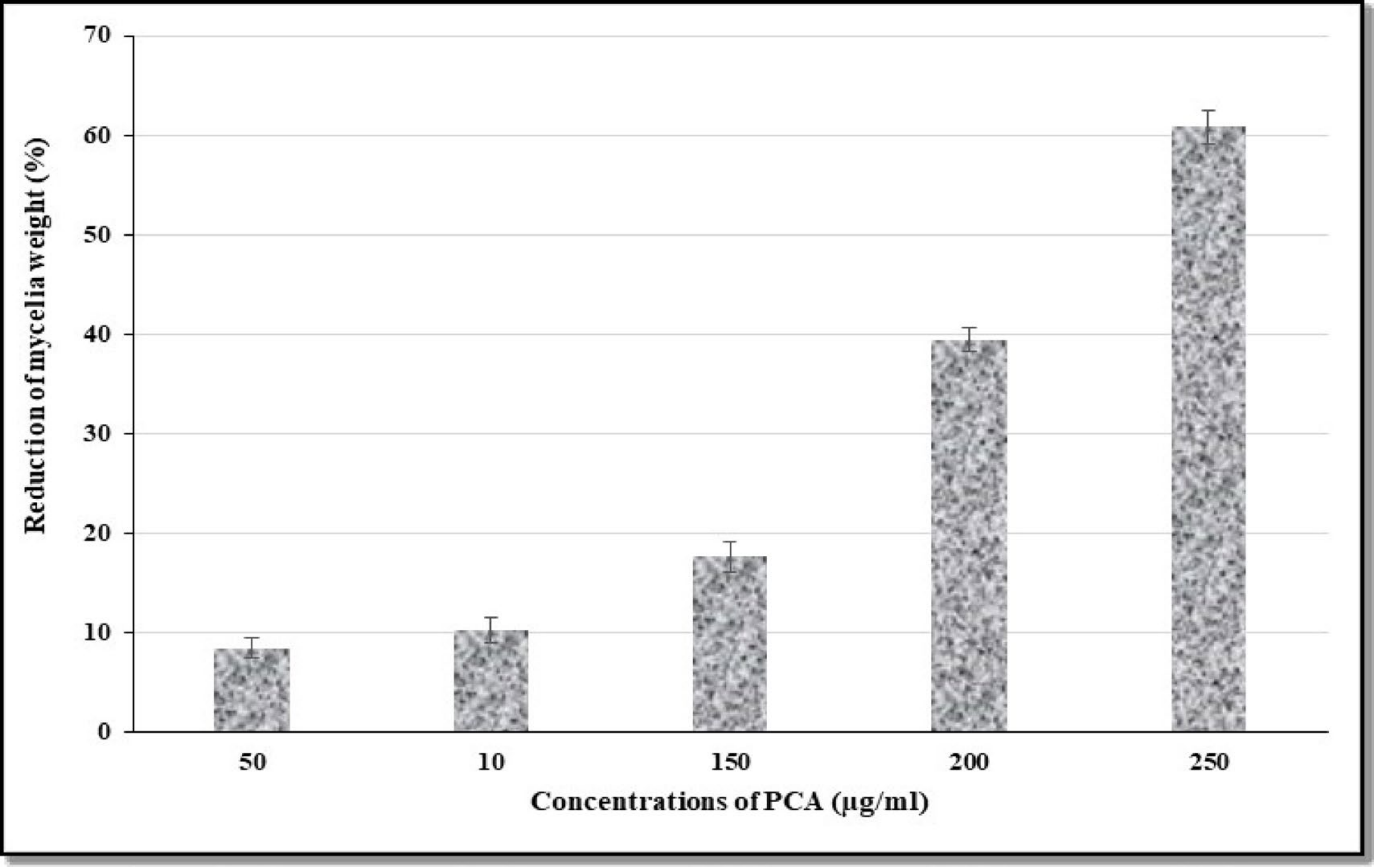
Effect of PCA concentration on biomass reduction of ***A. flavus*** in YES medium.

The results presented in Fig. 3 illustrate a clear dose-dependent relationship between PCA concentration and the reduction of AFB1 levels. As the PCA concentration increases from 50 mg/L to 250 mg/L, the percentage of AFB_1_ reduction significantly rises from 27 to 80.21%. This trend indicates that PCA effectively diminishes AFB_1_ production at higher concentrations. The reduction of 80.21% at the highest concentration (250 mg/L) is particularly noteworthy, suggesting that PCA could serve as a potent natural agent for lowering AFB_1_ contamination, which is critical for food safety. Both the Polynomial and Logarithmic models fit the data well, as shown in Fig. 4, confirming the consistency of this effect. The mechanism underlying PCA’s reduction of AFB_1_ may involve interference with key enzymes in the aflatoxin biosynthetic pathway, such as polyketide synthase, which is essential for aflatoxin synthesis^27–29^. Additionally, PCA reduced the mycelial weight of *A. flavus* to 60.8% of the control at a concentration of 250 µg/ml, as illustrated in Fig. 5. This reduction in fungal growth could be attributed to PCA-induced denaturation of proteins, inhibiting enzyme systems necessary for mycelial development. Moreover, PCA may impede DNA replication upon treatment, further contributing to growth suppression^30,31^.

### Impact of PAC on AFB_1_ biosynthesis and transcription of biosynthetic genes in *Aspergillus flavus*

**Fig. 6:**
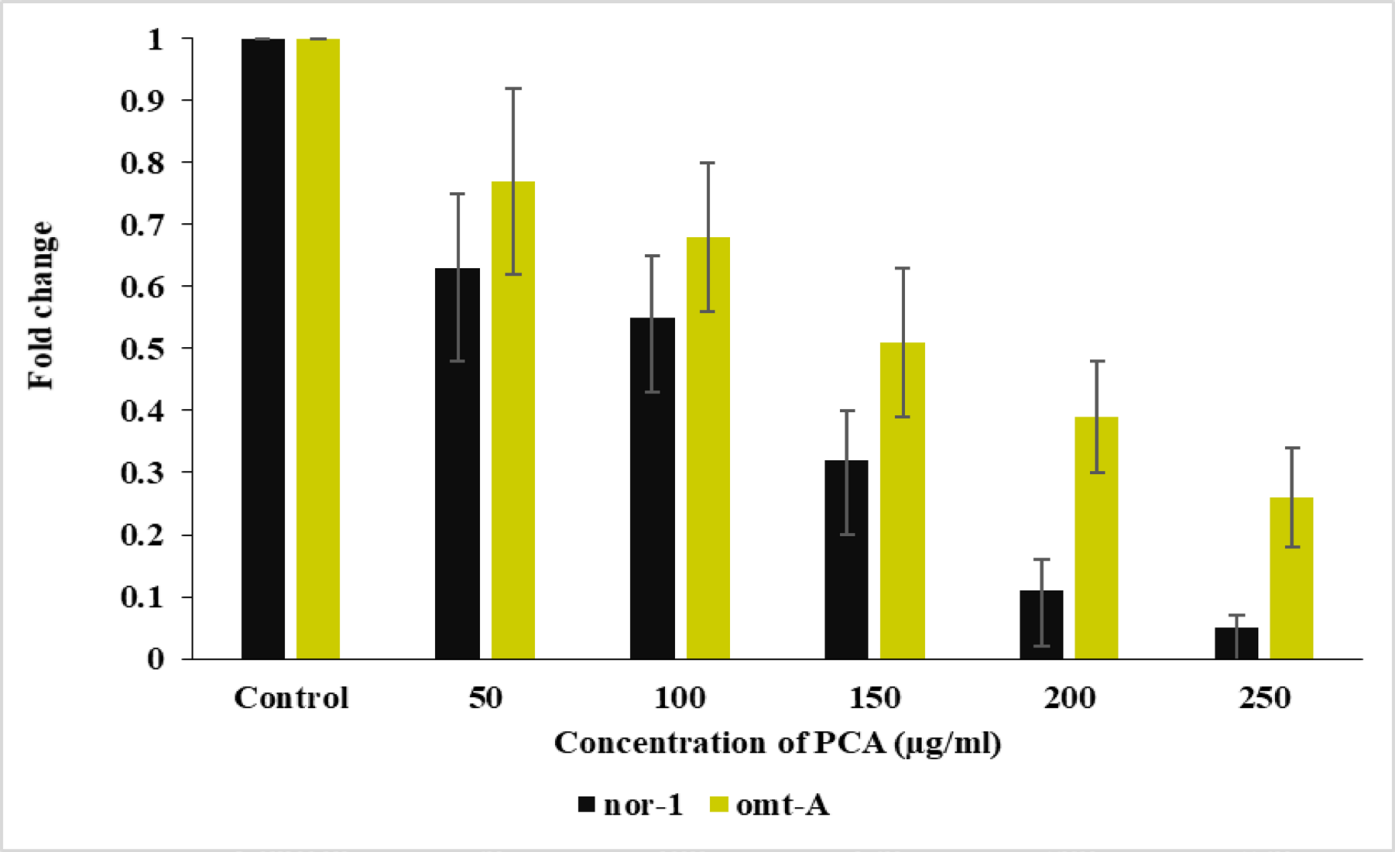
The impact of PCA on the transcription levels of ***nor****−1* and ***omt-A*** genes in ***A. flavus***.

The genes *nor-1* and *omt-A* are critical components of the AFB_1_ biosynthetic pathway in *A. flavus*. These genes encode enzymes that facilitate the conversion of intermediates into aflatoxins, making their transcription essential for AFB_1_ biosynthesis. The results indicated that at a concentration of 250 µg/ml of PCA, gene transcription was reduced by 95% for *nor-1* and by 74% for *omt-A*, respectively (Fig. 6). The data indicate a dose-dependent inhibitory effect of PCA on the transcription of the *nor-1* and *omtA* genes, which are involved in AFB_1_ biosynthesis. Both genes show a marked decline in transcription as PCA concentration increases, with *nor-1* being more sensitive to PCA than *omt-A*. This suggests that PCA may interfere with the aflatoxin biosynthetic pathway at multiple points, but particularly at earlier stages (as *nor-1* is involved in early steps of aflatoxin synthesis)^32–34^. On the other hand, *omt-A*, also known as *aflP*, is responsible for converting O-methylsterigmatocystin to AFB_1_, one of the final steps in the pathway. Although *omt-A* transcription is also downregulated by PCA, its inhibition is less pronounced than that of *nor-1*, particularly at lower concentrations of PCA. This suggests that while PCA impacts the entire biosynthetic pathway, its effect on the earlier steps (via *nor-1*) is more potent than on the later stages (via *omt-A*)^35–38^. Numerous studies have shown that many phenolic compounds and similar bioactive molecules can effectively inhibit AFB_1_ biosynthesis by modulating the expression of key regulatory and structural genes within the aflatoxin biosynthetic cluster. For instance, the regulatory genes *aflR* and *aflS* encode transcription factors essential for activating the entire aflatoxin gene cluster, and their downregulation has been closely linked with reduced aflatoxin production^26^. Several studies have reported that phenolic compounds such as curcumin, quercetin, and resveratrol significantly suppress the expression of *aflR* and *aflS*, thereby decreasing downstream structural gene expression including *nor-1*, *omt-A*, *aflM*, and *aflO*^26,33,36,37,39^. These compounds appear to interfere with fungal transcriptional machinery and oxidative stress pathways, leading to a comprehensive inhibition of aflatoxin biosynthesis. Such multi-target modulation underscores the potential of natural antifungal agents like PCA to disrupt aflatoxin production by affecting both regulatory and biosynthetic genes, rather than acting on a single gene or step in the pathway.

Finally, although this study focused on *nor-1* and *omt-A*, which represent early and late-stage genes in the aflatoxin biosynthetic pathway, we acknowledge the importance of regulatory genes such as *aflR* and *aflS*, which coordinate expression across the gene cluster. Future studies should expand gene expression analysis to include these key regulatory elements, as well as intermediate biosynthetic genes such as *aflM* and *aflO*, to build a more comprehensive mechanistic framework for PCA’s anti-aflatoxigenic action.

### Anti-aflatoxin effect of PCA in postharvest corn grains

**Fig. 7:**
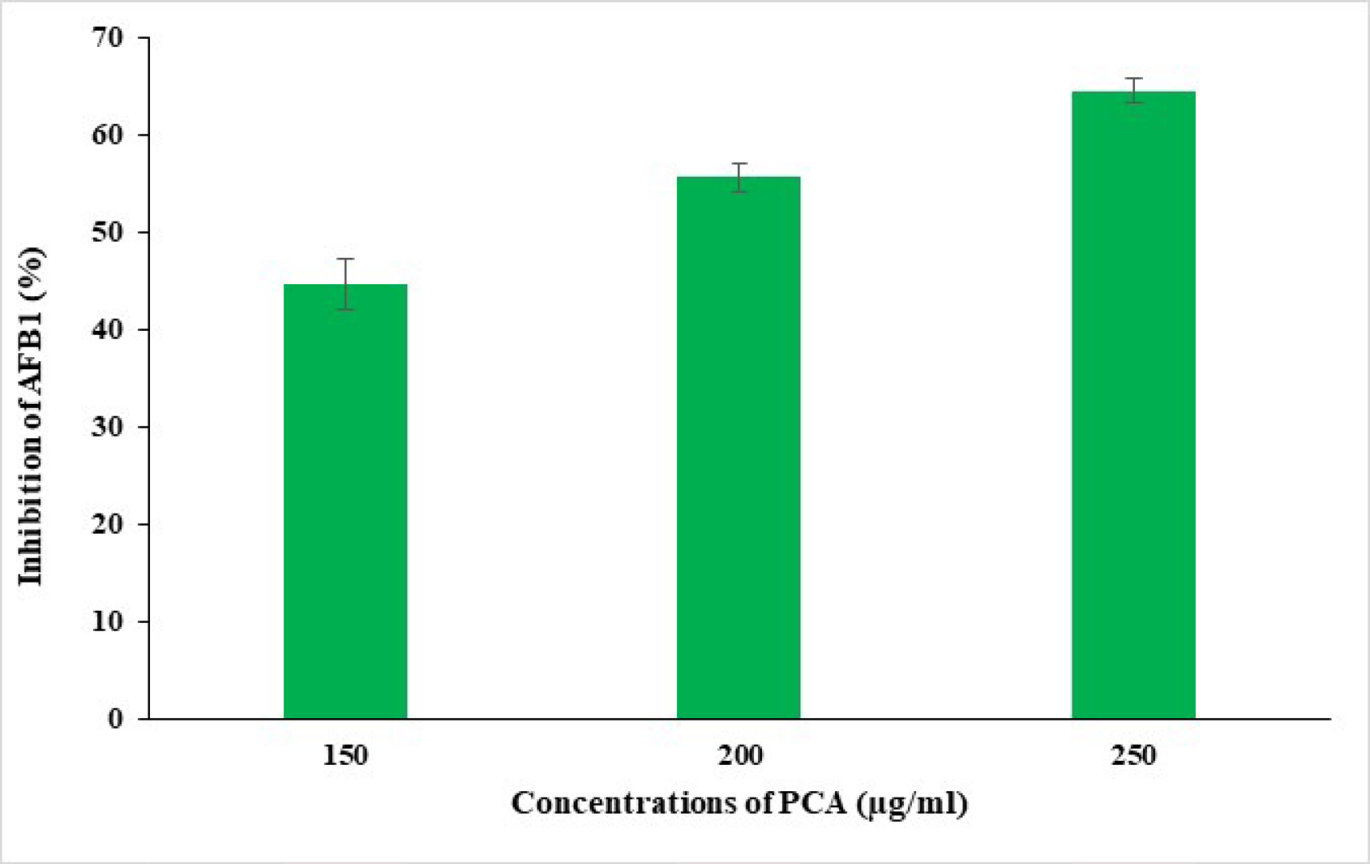
Efficacy of PCA in reducing AFB_1_ levels in corn grains.

Poor storage conditions, particularly concerning temperature and humidity, can result in substantial fungal growth on grains, leading to yield losses of 3–10% in developed countries and as much as 50% in certain developing regions. Spores of Aspergillus spp. are notably resilient, capable of easily dispersing through the air and remaining viable for extended periods^40,41^. This study aims to evaluate the efficacy of PCA in controlling AFB_1_ contamination caused by *A. flavus* on white corn grains. Data presented in Fig. 7 show that the percentages of AFB_1_ inhibition in corn were 44.8%, 55.7%, and 64.6% following treatment with PCA at concentrations of 150, 200, and 250 µg/ml, respectively. The findings suggest that PCA holds promise as a natural antifungal agent against AFB_1_ contamination in white corn grains. Its concentration-dependent inhibitory effect highlights its potential application in postharvest management practices to enhance food safety and reduce health risks associated with aflatoxin exposure. Further research is essential to fully understand its mechanisms of action and optimize its use in agricultural and food processing contexts.

## Conclusion

This study demonstrates that PCA exhibits significant antifungal and anti-aflatoxin properties against mycotoxigenic fungi. Notably, PCA effectively reduced AFB_1_ production in liquid media, achieving a maximum inhibition rate of 80.21% at a concentration of 250 µg/ml. This reduction indicates that PCA has the potential to target and inhibit critical enzymes within the aflatoxin biosynthetic pathway, thereby preventing AFB1 contamination, which is vital for ensuring food safety. Additionally, PCA was shown to significantly downregulate the expression of essential biosynthetic genes, *nor-1* and *omt-A*, with a more pronounced effect on *nor-1*. This finding suggests that PCA interferes with the aflatoxin production process at various stages, particularly during the early phases of synthesis. The study also highlights the effectiveness of PCA in controlling AFB_1_ levels in postharvest corn grains, illustrating its potential application in agricultural practices aimed at combating mycotoxin contamination. By emphasizing the concentration-dependent inhibitory effects of PCA, this research supports its viability as a natural agent for enhancing food safety and mitigating health risks associated with aflatoxin exposure. Future research should focus on further elucidating the mechanisms underlying PCA’s antifungal and anti-aflatoxin effects, as well as optimizing its applications in agricultural and food processing settings.

## Data Availability

All data supporting the findings of this research are available within the article.
